# Community Health Workers. Reinforcement of an Outreach Strategy in Rural Areas Aimed at Improving the Integration of HIV, Tuberculosis and Malaria Prevention, Screening and Care Into the Health Systems. “Proxy-Santé” Study

**DOI:** 10.3389/fpubh.2022.801762

**Published:** 2022-02-24

**Authors:** Desmorys Raoul Moh, Maika Bangali, Patrick Coffie, Anani Badjé, Adahourman Aman Paul, Philippe Msellati

**Affiliations:** ^1^Unité Pédagogique de Dermatologie et Infectiologie, UFR Sciences Médicales, Abidjan, Côte d'Ivoire; ^2^Programme PAC-CI, Abidjan, Côte d'Ivoire; ^3^Inserm 1219, Université de Bordeaux, Bordeaux, France; ^4^Direction Départementale de la santé, Toumodi, Côte d'Ivoire; ^5^UMI TransVIHMI, Institut de Recherche pour le Développement, Abidjan, Côte d'Ivoire; ^6^Université de Montpellier, Montpellier, France

**Keywords:** community health workers, capacity building, outreach strategies, rural areas, Côte d'Ivoire, HIV, malaria, tuberculosis

## Abstract

**Background:**

In Côte d'Ivoire, the health system remains poorly accessible and inefficient, particularly in rural areas. Malaria, tuberculosis and HIV remain a major concern. Tasks shifting to Community Health Workers (CHWs) in rural areas has been proposed in terms of responses and has shown encouraging results with some limitations. Objective is therefore to develop and implement, in a health district, at the level of a neighborhood, a sub-prefecture, two villages and two camps, innovative strategies aimed at improving the integration of HIV, malaria and tuberculosis prevention and care into the health system at the community level through CHWs.

**Methods:**

Introduce innovations to be integrated into the national system: (i) Selection and strengthening of the capacities of CHWs to provide care for the three diseases through home visits [Information Education and Counseling/Communication for Behavior Change (IEC/CBC)], simple malaria screening and management, referral of suspected tuberculosis cases and Directly Observed Treatment, short-course (DOTS), screening, prophylaxis and distribution of antiretrovirals (ARVs) to HIV-infected patients; (ii) monthly animation of village health committees by target groups (women of childbearing age, children under 5 years old, young adolescents); (iii) use of an application and tablets for data collection.

**Discussion:**

This innovative project integrates new activities such as ARV distribution by CHWs, management of pre-exposure prophylaxis in rural areas and electronic data capture by communities. Several lessons can be learned on the relevance of the role and activities to be carried out by these CHWs in the fight against these three diseases.

**What is already known on this subject?**

In Côte d'Ivoire, community health workers (CHWs) have proven to be effective in vaccination campaigns as well as in major endemics control, such as malaria; however, there is very few action on their part in terms of offering HIV testing, tuberculosis screening, and community distribution of antiretrovirals and antituberculosis drugs.

**What does this study add?**

This study allows for an integrated offer of care in a context of new information and communication technologies, while efficiently building the capacity of CHWs.

They will be perfectly able to contribute to the HIV and tuberculosis control on a routine basis, and can represent, in certain situations, the first level of contact of the population with health care. In addition to that, with a minimum of technical means, it is possible to have health data from the field in real time.

## Policy Implications

In a context of limited access to care for these populations in rural areas (distance from the health center, costs, insufficient personnel), the use of CHWs properly trained in diagnosis and minimal care, supervised and well-paid, is a solution for access to care for all.

## Introduction

Community health workers (CHWs) are a critical link in promoting healthy behaviors in populations and improving health systems around the world. Over the past decade, evidence has emerged regarding CHWs and their potential to improve population health where health workforce resources are limited and access to basic services is low (mainly in low-income countries) (1). In low-income countries, CHWs can make major improvements in priority health areas, including reducing child undernutrition, improving maternal and child health, increasing access to family planning services, and contributing to the fight against HIV, malaria, and tuberculosis (1).

Progress has been made in the detection, treatment and prevention of major communicable diseases such as HIV, malaria and tuberculosis (2). However, further work is needed to achieve the 2015–2030 Sustainable Development Goals (SDG). SDG 3 focuses global attention on infectious diseases with the goal (3.3) to halt the epidemics of AIDS, tuberculosis, malaria and neglected tropical diseases by 2030 and to combat hepatitis, waterborne and other communicable diseases (2).

In low- and middle-income countries, community engagement initiatives have been described as “critical enablers” in the response to communicable diseases. Such initiatives can be particularly important in contexts where health systems are under-resourced, and communities become a key and important resource for achieving behavior change (3–8).

Côte d'Ivoire is a country in the Gulf of Guinea with a population of more than 22 million in 2014 (9) of which eighteen percent of the population is under 5 years old and more than 5 million women are of childbearing age. The country is showing fairly significant economic growth, but wealth is unevenly distributed (49% of the population lives below the poverty line). This poverty is especially present in rural areas where it can affect more than 60% of the population.

Health indicators do not track the country's relative prosperity. What could be called the “Ivorian health paradox” is expressed both by relatively high coverage in health structures and poorer health indicators. Maternal mortality, which is one of the best indicators of a country's health status, is very high and accounts for 614 deaths per 100,000 live births, placing Côte d'Ivoire in 6th position (out of 15) of the worst maternal death rates in West Africa. The situation is similar with infant and child mortality, which represents 125 per thousand live births.

With regard to the three pandemics against which the Global Fund is mobilizing, malaria still accounts for 35% of consultations in health facilities in 2016. Malaria mortality is 96‰. In 2017, we were at 3,332 deaths per year from malaria. In 2020, we were at 1,641 deaths. That is to say a mortality rate down by about 50%. And this regression continues in the same year.

HIV remains a major concern even though prevalence has declined significantly over the last 10 years, with a prevalence of 2.9% in 2017 among people aged 15–64. Treatment with antiretroviral drugs has led to considerable progress and apllied to more than 200,000 people in 2018. However, the challenge of screening remains major (10).

Finally, tuberculosis is still present in Côte d'Ivoire. In 2018, the incidence was 84 cases per 100,000 inhabitants (new cases and relapses all forms of location combined). According to the 2018 annual report of the National Tuberculosis Control Program (NTCP), 21,301 new cases were reported, 68% of which were of the contagious form (bacteriologically confirmed pulmonary tuberculosis) (11).

The overall objective of this project is therefore to develop and implement, in a health district, at the level of a neighborhood, a sub-prefecture, two villages and two priority camps, innovative strategies to improve the integration of HIV, malaria and tuberculosis prevention and care into the health system at the community level through CHWs.

We hypothesize that the action of CHWs will improve the health of the populations concerned (pregnant women, infants, children, adolescents and youth) in the area of the three pandemics that the Global Fund is fighting (malaria, tuberculosis and HIV) as well as in the area of maternal and child health.

## Methods

**Figure 1:**
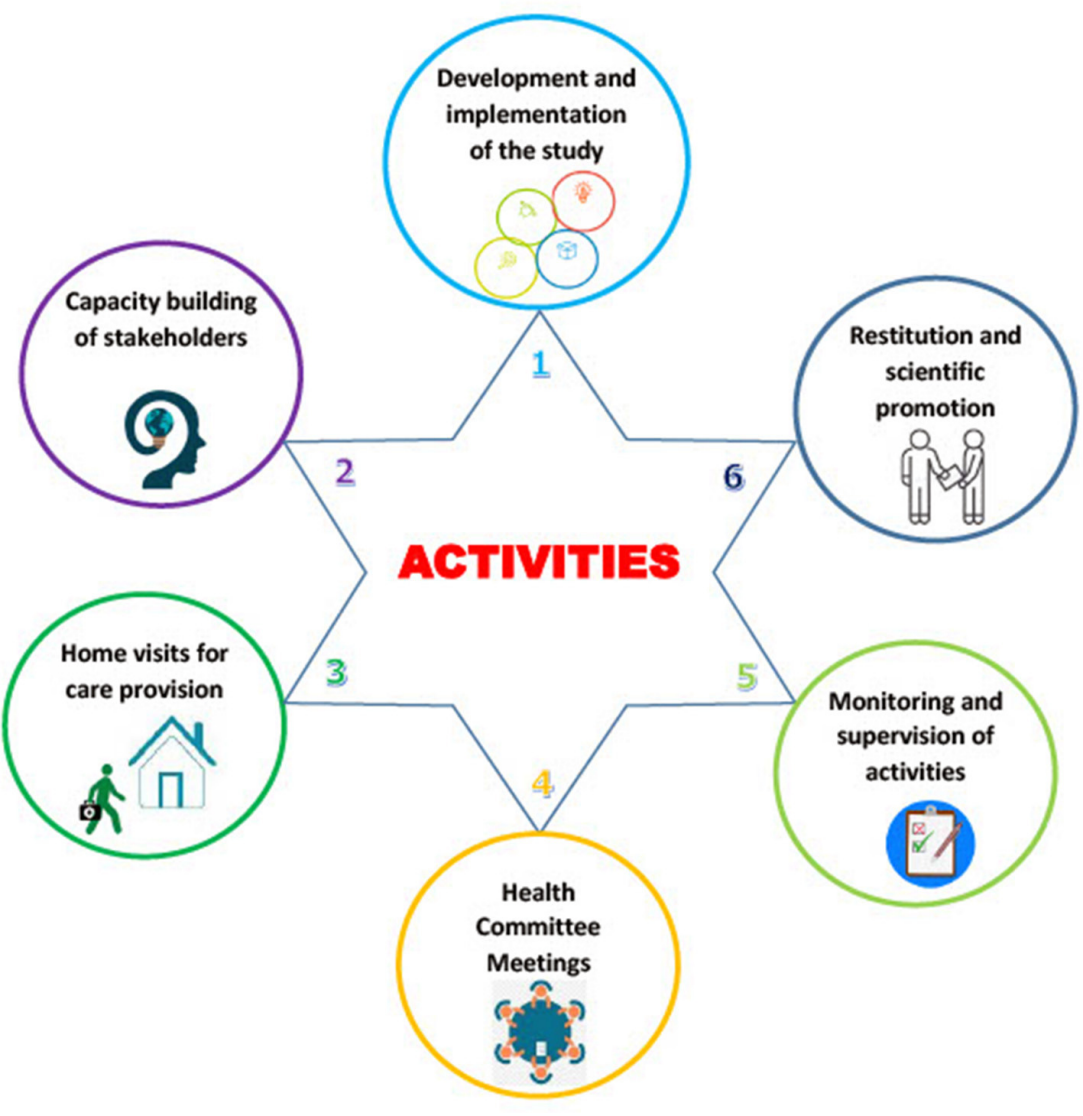
Main activities of the ≪ proxy santé ≫ study.

This project is planned for a duration of 3 years, including 2 years of field activities with six main activities (all summarized in Figure 1).

### Activity 1: Development and Implementation of the Study

We chose a department called Toumodi located in the ≪ Bélier ≫ region at 200 kms from the city of Abidjan, the economic capital of Côte d'Ivoire and 46 kms from Yamoussoukro, the political capital. It has a population of 42,000 inhabitants. The sub-prefecture is Djekanou. With a population of 21,000 inhabitants, it is the chief town of the commune. Kokoumbo and Kpouebo, the two localities of this sub-prefecture chosen for the intervention of the CHWs, have 25,000 inhabitants each. These three localities are home to the largest active number of people living with HIV in the department.

The beginning of the activities will be preceded by meetings with the health authorities and a health Non-Governmental Organization (NGO) to present the project to them and to identify the actors (nurses, members of the district management team and CHWs) who will be involved in the activities.

In addition, spots will be broadcasted on local radio stations to sensitize the population, assure them of the support of the health and community authorities and facilitate home visits.

A launching ceremony will mark the beginning of the activities to which will be invited the populations as well as the health care teams and the administrative authorities.

An electronic application will be developed and should be compatible with the existing information system at the national level to facilitate the transfer of data collected and thus integrate the health system. The data circuit will be developed so as not to create a parallel circuit. Thus, field data will be synchronized on a server located in the Toumodi Health District.

The data collected by CHWs will be done using touch-sensitive tablets. CHWs will receive appropriate training in the use of the tablets. Data will be saved on a daily basis and uploaded to a server automatically at the Health District after verification by the nurse of the corresponding health center, using a 3G flash disk. The server administrator will check the quality of the data with automatic procedures and will export them to a server located at the monitoring and management center located at the PAC-CI program in Abidjan. The acceptability of the use of these tablets and especially the feasibility of using such a technology in remote rural areas will be studied. CHWs will be invited to report any problems and difficulties of use on a forum dedicated to this exchange.

The data collected will be used to complete the medical records of the people monitored by the CHWs.

The questionnaire installed on the tablet will be composed of a “household description” section and individual records for each member of the household. The individual files will be constructed for the different populations concerned: adult men, adolescent and young men, adult women, adolescent and young women, children under 5 years old and children between 5 and 10 years old.

### Activity 2: Capacity Building of Stakeholders

Twenty-four CHWs will be designated by their community. They will be trained in partnership with the Community Health Departement. The trainers are members of the national pool of CHW trainers and training modules are available for the management of Malaria, HIV and Tuberculosis by community workers. The trainings include a theoretical component and a practical component with field coaching.

Supervision visits will make it possible to evaluate the quality of the services offered and to take corrective and remedial actions.

They will then be deployed in the field, covering two neighborhoods in the town of Djekanou (10 CHWs), the localities of Kokoumbo (5 CHWs) and Kpouebo (5 CHWs) and 4 surrounding camps (1 CHW per camp). These camps will be drawn at random from the list of camps located around the two localities of Kokoumbo and Kpouebo.

### Activity 3: Home Visits for Care Provision

The project provides initial home visits during which the CHW will be responsible for making an inventory of the different components of the household (number of people, gender, age, vaccination status of children under 5 years old, carrying out prenatal consultations for pregnant women, post-natal visits, etc.). These initial visits may take place in a maximum of three times depending on the availability of household members. After the initial visit, the following visits will be monthly home visits known as reinforcement visits in order to (i) inquire about news from the family and collect any changes or developments since the last visit (pregnancy, birth, intercurrent pathology, etc.), (ii) ensure follow-up of people under treatment (adherence, changes in health status, etc.).

During the home visits, each CHW will be expected to provide services in the fight against malaria, HIV and tuberculosis in 50 households. These households will be enumerated and drawn at random in close collaboration with a statistician from the National Institute of Statistics.

#### Malaria Prevention and Management

- Facilitate IEC and CBC sessions on environmental health (destruction of larval deposits).
- Raise awareness about the use of LLINs (Long Lasting Insecticide Treated Nets), distribute the nets to families including pregnant women and children under 5 years of age, and demonstrate the correct installation of the nets and their proper use.
- Make rapid diagnostic tests (RDTs) available to CHWs to test for malaria in any febrile individual.
- Training in the recognition of the signs of simple or severe malaria and management of simple malaria cases by the administration of anti-malarial drugs based on Lumefantrine and Artemether or other combinations according to current recommendations.
- Refer severe cases as well as non-malarial fevers (severe malaria and fevers with negative rapid malaria screening test) to the nearest health center.

#### Tuberculosis Prevention and Management

- Systematic screening for tuberculosis [interview and search for four symptoms (cough, fever, weight loss, night sweats)] with referral of suspected cases (those with these symptoms) to the nearest management center for sputum examination.
- If tuberculosis is confirmed and treated: support for adherence to treatment and implementation of community-based DOTS (Directly Observed Treatment).
- Ensure that children under 5 years of age in the index case setting receive isoniazid (INH) TB prophylaxis and that TB-HIV co-infected individuals not yet on ARVs have access to INH prophylaxis.

#### HIV Prevention and Management

- Promoting HIV testing for all household members and offering counseling and testing to volunteers.
- In case of positivity, referral to the nearest treatment center.
- As part of the follow-up:

° Support for adherence to treatment
° Tuberculosis screening
° Offers screening to spouses and children
° Proposal of pre-exposure prophylaxis (PREP) for serodifferent couples (as a pilot activity)
° Facilitation of the delivery of ARVs to patients

In total, these CHWs will each intervene with 50 households, or 1,200 households in total, representing about 12,000 people. These 12,000 people are composed of about 6,000 children under 15 years old and 6,000 adults.

### Activity 4: Health Committee Meetings

CHWs will be responsible for the animation of village health committees. These committees will meet monthly by target groups: groups of women of childbearing age and children under 5 years old and groups of youth and adolescents (2 meetings/month/CHW). These meetings will be an opportunity to sensitize and, depending on the target, address the following themes:

- Sexual and reproductive health,
- Family planning,
- Environmental hygiene,
- Correct use of LLINs
- Vaccinations,
- Breastfeeding,...

Ideally, it should be possible to film these meetings if the people attending agree. The content will be saved and analyzed by a social scientist (anthropologist or sociologist) to see if the content disseminated by the CHWs is correct and relevant, and also to analyze the participants' statements in order to identify reflections, obstacles and demands of the community. The social scientist will participate in the scientific committee and in the third year will prepare a report on this component of the activities and the information that he (she) has been able to draw from it.

In addition to these operational studies targeting these three pathologies and included in the intervention, specific investigations will be conducted:

- The feasibility of disclosing HIV+ status among sero-different couples and the acceptability of sharing this status with CHW will be studied.
- The feasibility of pre-exposure prophylaxis (PREP) in the negative member of serodiscordant couples in rural areas and its impact on the sexuality of the couple and the sharing of serological status will also be studied.
- The use of antiretrovirals in both partners and the difficulties encountered will be documented. A specific questionnaire will be developed and administered on a monthly basis by the CHW. It will also be analyzed on a monthly basis, in order to document and correct any difficulties that may arise.
- Another specific survey will concern the distribution of ARVs in the community setting: implementation of a community ARV collection system in response to difficulties in accessing health facilities. Transportation costs and time spent are elements that can lead to non-regularity in monitoring. The major issue will be not only feasibility, which is a real challenge, but also confidentiality and the acceptability of status sharing. The CHW will collect information specific to this intervention and will share the data collected with the professionals during the follow-up meetings. A specific monthly analysis will document and correct any difficulties.
- We estimate, per year, in a rural area such as this one, with 12,000 inhabitants monitored, four deaths of pregnant women or in the immediate post-natal period (within 40 days), a little more than 350 deaths of children under 5 years of age, about 30 cases of HIV infections (half of which are monitored), <20 confirmed cases of tuberculosis and about 1,000 cases of malaria. The project will make every effort to ensure that in the localities where the project is being implemented:
- The number of severe malaria cases is reduced by 50%.
- The number of malaria-related deaths is reduced by 50%;
- 100% of chronic coughers diagnosed with tuberculosis are referred and managed by the health system;
- 100% of confirmed tuberculosis patients monitored by CHWs are observant and have completed their treatment.
- 100% of people who test positive for HIV initiate ARVs.
- 90% of HIV-positive people are maintained in care
- 90% of scheduled home visits are completed.

### Activity 5: Monitoring and Supervision of Activities

A centralized monitoring system will allow the systematic collection of information that could be used to determine the progress of the project on an ongoing basis, but also the difficulties encountered. This will be done through the evaluation of the quality of the data transmitted by the tablets and ensured by the database manager through well-defined quality indicators. It will also be done through visits to the project implementation sites. These visits will be made monthly for the first 3 months for an earlier detection of possible difficulties and then every 2 months thereafter.

### How Were Households Selected?

Using a map of the region obtained from the National Institute of Statistics, each CHW was assigned an “enumeration area” (EA). They were thus able to systematically enumerate all the households in their EA and identify which of these households were eligible (at least one child under 5 years old and/or a pregnant woman) and which were not. A total of 3,301 households were identified within a 5 km radius of the health center in each locality, 71% of which were eligible for the study.

## Discussion

This community participation project aims to improve the population's access to Primary Health Care services and to restore users' confidence in public health services. Despite the Ivorian authorities' stated commitment, the health system is not very accessible, particularly in rural areas, and therefore not very efficient. It remains essentially the responsibility of households and often represents so-called “catastrophic” health expenditures. In terms of response, this situation has led to a willingness to rely on a certain tasks shifting in the health system, a greater focus on prevention activities and the need to rely on the communities themselves.

The aim here is to create community relays, such as Community Relay Agents within the communities. This deployment of Community Health Workers (CHWs) is already the subject of implementation documents at the level of the Ministry of Health of Côte d'Ivoire and its Community Health Department. It has been carried out for several years but with limitations related to the not always perfectly defined status of these CHWs, the heterogeneity of their training, the problem of their remuneration, and their situation within the health system.

Faced with poor health indicators, the Ministry of Health and Public Hygiene of Côte d'Ivoire has for several years chosen to rely on the community. Our project is therefore complementary to the community-based program to fight AIDS, tuberculosis and malaria financed by the Global Fund in Côte d'Ivoire in several ways:

- We have been careful not to duplicate what is already being done under the Global Fund and we would like to introduce innovations that could then be integrated into a proposal to the Global Fund.
- This project contributes to improving access to prevention and care for fragile populations such as pregnant women, children, adolescents and young people who are the Global Fund's preferred targets. It also aims to strengthen the quality of services of health care structures in rural areas involved in the implementation of Global Fund grants.
- In addition, the introduction of new communication technologies such as tablets connected to a server should improve patient referral and the transfer of health information to the health structures concerned and to the departmental directorate of community health.

This project focuses on the role of CHWs in the fight against HIV, tuberculosis, and malaria. From this study, therefore, in view of the activities carried out by the CHWs and the results obtained, several lessons will be drawn on the relevance of the role and activities to be carried out by these CHWs in the fight against these three diseases. These results and lessons learned will be shared with the CHWs to obtain their involvement in the strengthening and improvement of these interventions.

During the project, meetings will be organized every 2 months in order to exchange with the different partners. Similarly at the end of the project, a restitution workshop will be organized with all the institutions and partners involved in the fight against the three diseases in order to discuss the results and lessons learned from this study and to see together how to use them and integrate them into national strategies. The contribution of these interventions to the improvement of their health by making the link with the socio-economic impact of a good health status will also be presented to them.

In order to ensure the sustainability of the interventions, a strong community commitment will be sought.

When CHWs are well-selected, trained and supervised by qualified individuals, and when adequate equipment, medications and diagnostic tools are properly made available to them within the framework of well-defined protocols, they contribute significantly to the improvement of key and primordial health-related behaviors. They also contribute to the strengthening of links between communities and health services and to improving access to key health services. CHWs should therefore become an integral part of health systems as they strive to improve their quality, coverage and impact on the health of the population (1).

CHWs should not be a separate and autonomous entity within the health system. They are an essential element in activities involving specific interventions within the framework of quality, community-based service delivery. Without the support of communities and health systems in a collaborative setting (to train them, supervise them, select appropriate technical interventions and provide the necessary logistics), interventions would suffer (1).

One of the key challenges remains strong institutional support, sustainable financial support in order to ensure sustainability beyond the funding of these activities through research projects.

Health is a human right, defined by the World Health Organization (WHO) as “a state of complete physical, mental and social wellbeing and not merely the absence of disease or infirmity”. This project will contribute to the improvement of the health status of the populations in the intervention zone, which will enable them to go about their rural or commercial activities more serenely and thus be able to take better care of themselves. These interventions will thus be able to promote the physical, mental and social development of the beneficiaries and extended to other rural areas of the country and could serve as models for other countries in Sub-Saharan Africa.

This study would be “futile” without a policy of disseminating the results. To this end, the results of the project will be presented at the level of the three localities as well as at the central level in Abidjan: a capitalization and perspectives seminar will be organized to conclude the 3 years of work and to start the “after” of the project.

As part of the link with the health system, an information system will be developed and set up to study the feasibility of transferring community data into the national health information system.

## Ethics Statement

The studies involving human participants were reviewed and approved by National Committee of Life Sciences and Health of Côte d'Ivoire (CNESVS). Written informed consent to participate in this study was provided by the participants' legal guardian/next of kin.

## Author Contributions

DM is the principal investigator of the study and cowrote this manuscript. MB, PC, and AB cowrote this manuscript. PM validated the manuscript. All authors contributed to the article and approved the submitted version.

## Funding

This study was funded by Expertise France-Initiative 5% (610.000 euros for 3 years). The funder ensures the total financing of the study over a period of 3 years: the salaries of the staff involved in the study, coordination costs, study activities (development and implementation of the study, purchase of equipment, capacity building of stakeholders, financing of home visits, animation of health committees, monitoring and supervision of activities, scientific valorization).

## Conflict of Interest

The authors declare that the research was conducted in the absence of any commercial or financial relationships that could be construed as a potential conflict of interest.

## Publisher's Note

All claims expressed in this article are solely those of the authors and do not necessarily represent those of their affiliated organizations, or those of the publisher, the editors and the reviewers. Any product that may be evaluated in this article, or claim that may be made by its manufacturer, is not guaranteed or endorsed by the publisher.

## Abbreviations

AIDS: Acquired ImmunoDeficiency Syndrome
ARVs: Antiretrovirals
CBC: Communication for Behavior Change
CHW: Care Health Worker
CPN: Prenatal Consultations
DOTs: Directly Observed Treatment, short-course
HIV: Human Immunodeficiency Virus
IEC: Information Education and Counseling
LLINS: Long Lasting Insecticide Treated Nets
NTCP: National Tuberculosis Control Program
PAC-CI: Programme National de Lutte contre le SIDA-Agence Nationale de Recherches sur le SIDA et les Hépatites Virales en France-Coopération Française- Côte d'Ivoire
PREP: Pre-Exposure Prophylaxis
RDT: Rapid Diagnostic Test
SDG: Sustainable Development Goals
WHO: World Health Organization.
